# Supplementary material for: Nuclear spin polarization of lactic acid via exchange of parahydrogen-polarized protons
Source: Commun Chem. 2024 Aug 8;7:172. doi: 10.1038/s42004-024-01254-8 (PMC11306230; doi:10.1038/s42004-024-01254-8)
Supplement: Supplementary file 2 — Supplementary Information [file 42004_2024_1254_MOESM2_ESM.pdf]

Supporting Information

for

Nuclear spin polarization of lactic acid via exchange of parahydrogen-polarized protons.

Kolja Them*[,1,2], Jule Kuhn[1], Andrey N. Pravdivstev[1] and Jan-Bernd Hövener*[,1,3]

[1]Section Biomedical Imaging, Molecular Imaging North Competence Center (MOIN CC), Department of Radiology and Neuroradiology, University Medical Center Schleswig-Holstein and Kiel University, Am Botanischen Garten 14, 24118 Kiel, Germany

*Corresponding author
[2]kolja.them@rad.uni-kiel.de
[3]jan.hoevener@rad.uni-kiel.de

Table of Contents

^{13}C-NMR SPECTRA OF $^{13}C_3$-LACTIC ACID HYPERPOLARIZED USING PHIP-X (PRECURSOR TO TARGET RATIO). 2

^{13}C-NMR SPECTRA OF $^{13}C_3$-LACTIC ACID HYPERPOLARIZED USING PHIP-X (MAGNETIC FIELD DEPENDENCE). 6

^{13}C-NMR SPECTRA OF ^{13}C-METHANOL HYPERPOLARIZED USING PHIP-X. 13

^{1}H-NMR SPECTRA OF ^{13}C-METHANOL HYPERPOLARIZED USING PHIP-X. 20

^{13}C-NMR SPECTRA OF HYPERPOLARIZED STYRENE IN THE PRESENCE OF METHANOL. 21

SPIN DYNAMICS SIMULATIONS. 22

^{13}C-NMR spectra of $^{13}C_3$-lactic acid hyperpolarized using PHIP-X (precursor to target ratio).

Note that it is possible to adjust the phase such that all LA-peaks show into the same direction, as in Fig. 3a of the main manuscript.

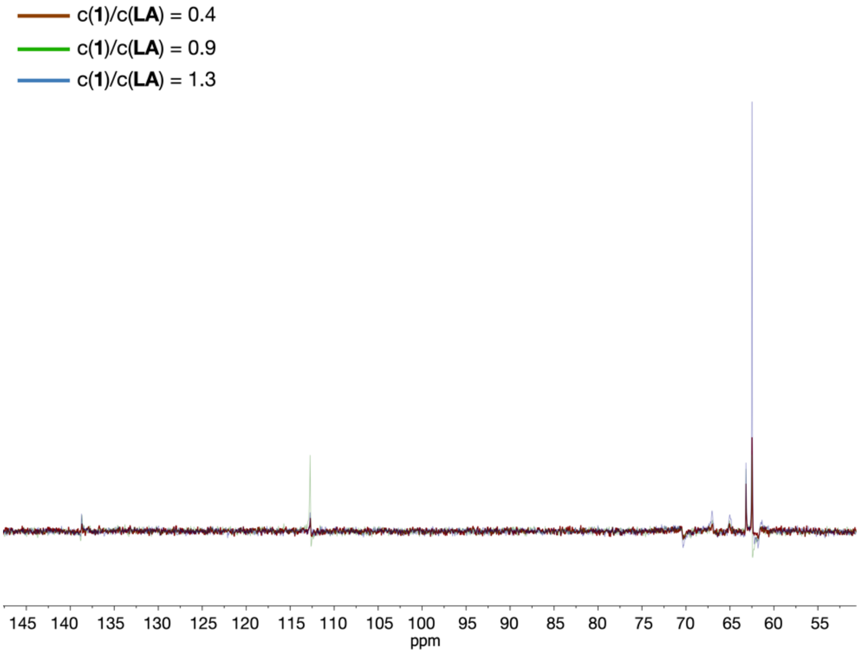

Figure S1: Superposition of ^{13}C-NMR spectra of $^{13}C_3$-lactic acid hyperpolarized using PHIP-X. The three corresponding PHIP-X experiments differ in c(1)/c(LA) =0.4 (brown line), 0.9 (green line) and 1.3 (blue line).

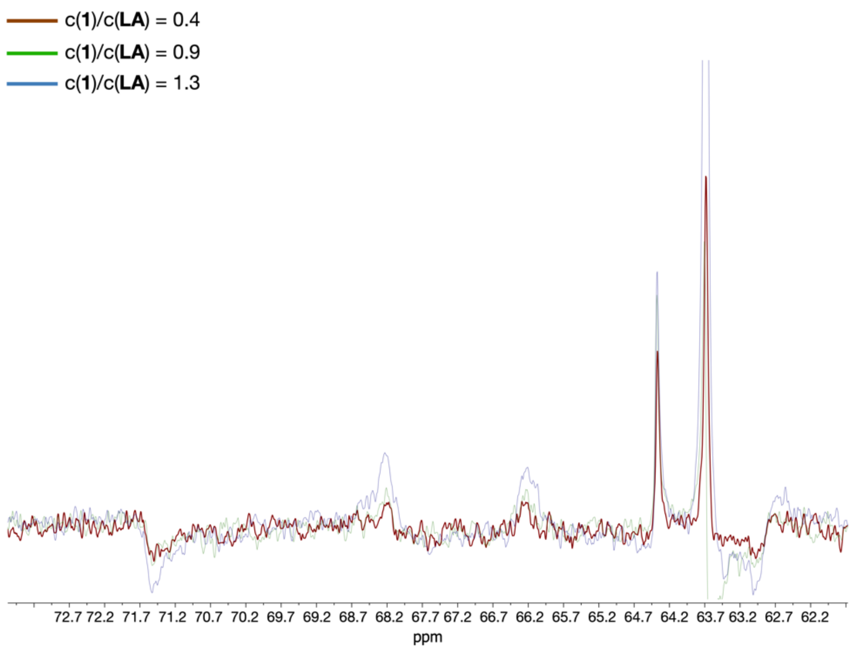

Figure S2: Superposition of ^{13}C-NMR spectra (zoom of figure S1) of $^{13}C_3$-lactic acid hyperpolarized using PHIP-X. The three corresponding PHIP-X experiments differ in c(1)/c(LA) =0.4 (brown line), 0.9 (green line) and 1.3 (blue line). The resonances at 66.2 ppm and 68.3 ppm were generated by the hyperpolarized 2-^{13}C nucleus of lactic acid.

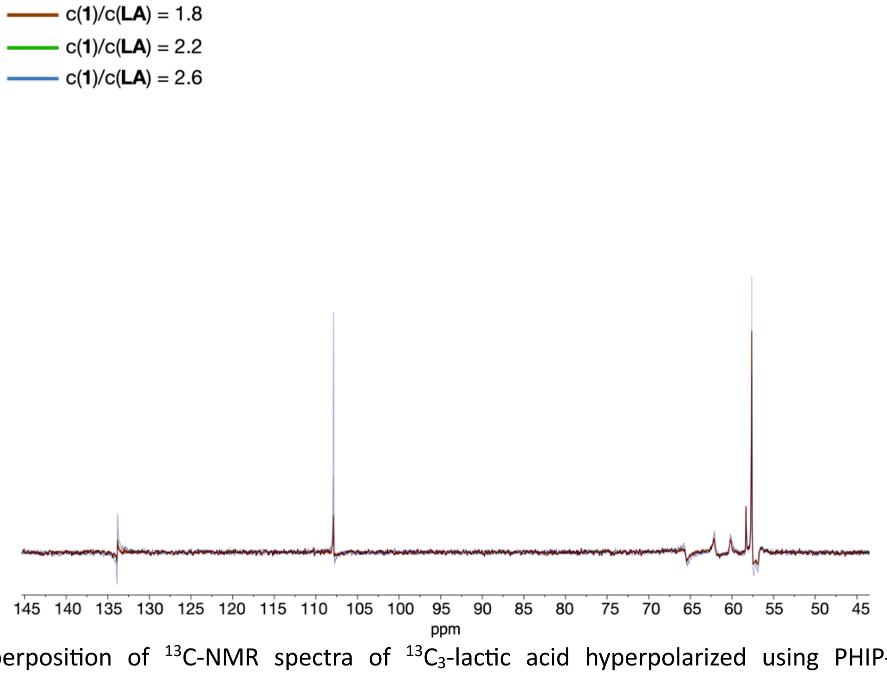

Figure S3: Superposition of ^{13}C-NMR spectra of $^{13}C_3$-lactic acid hyperpolarized using PHIP-X. The three corresponding PHIP-X experiments differ in c(1)/c(LA) =1.8 (brown line), 2.2 (green line) and 2.6 (blue line).

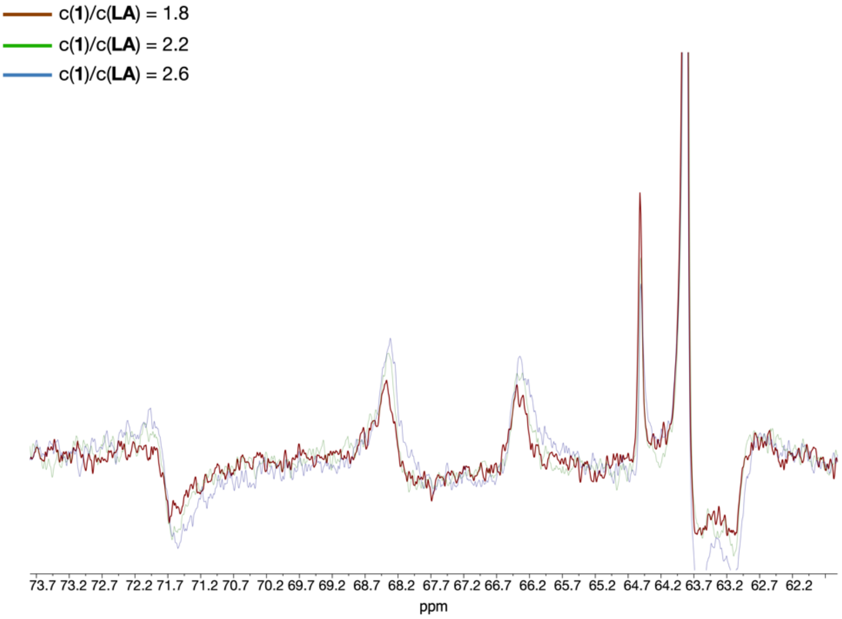

Figure S4: Superposition of ^{13}C-NMR spectra (zoom of figure S3) of $^{13}C_3$-lactic acid hyperpolarized using PHIP-X. The three corresponding PHIP-X experiments differ in c(1)/c(LA) =1.8 (brown line), 2.2 (green line) and 2.6 (blue line). The resonances at 66.2 ppm and 68.3 ppm were generated by the hyperpolarized 2-^{13}C nucleus of lactic acid.

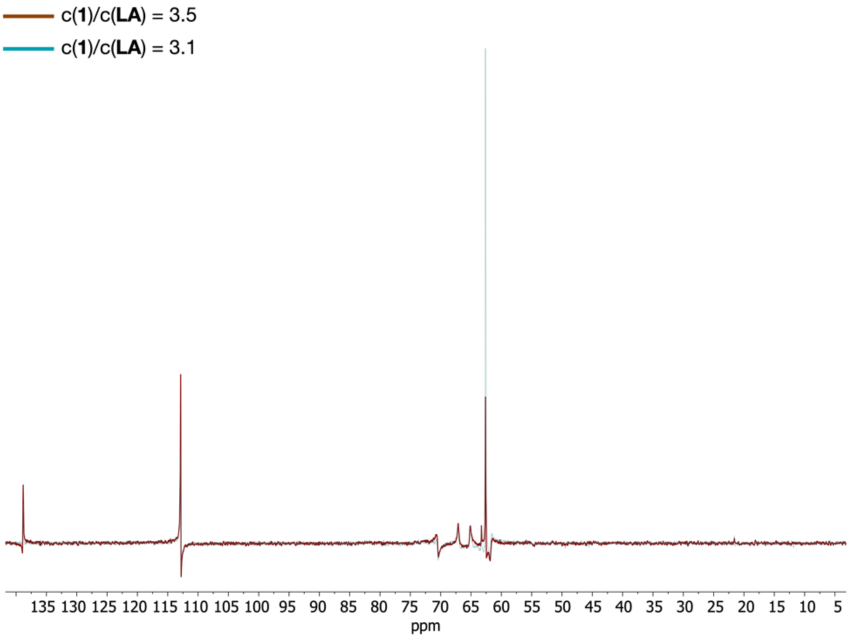

Figure S5: Superposition of ^{13}C-NMR spectra of $^{13}C_3$-lactic acid hyperpolarized using PHIP-X. The two corresponding PHIP-X experiments differ in c(1)/c(LA) =3.5 (brown line) and 3.1 (cyan line).

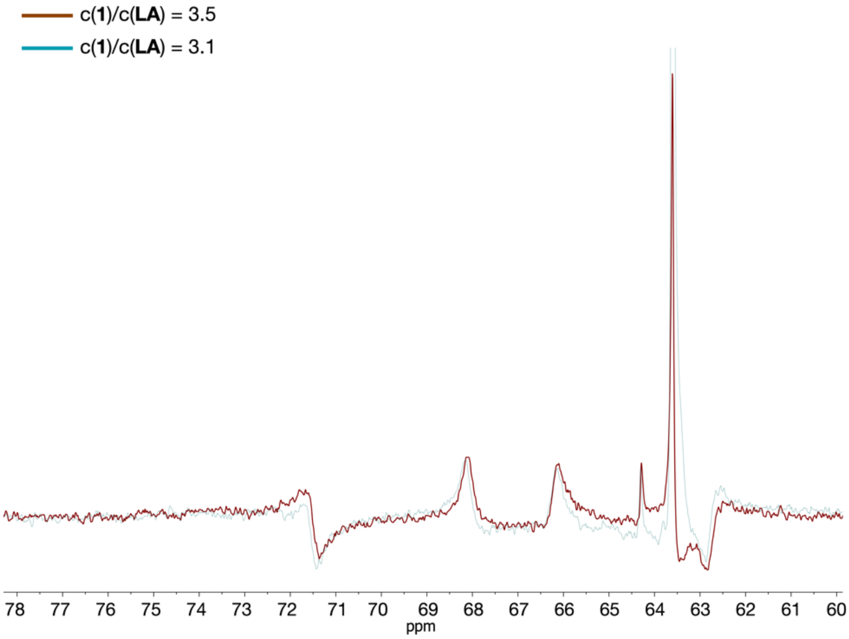

Figure S6: Superposition of ^{13}C-NMR spectra (zoom of figure S5) of $^{13}C_3$-lactic acid hyperpolarized using PHIP-X. The two corresponding PHIP-X experiments differ in c(1)/c(LA) =3.5 (brown line) and 3.1 (cyan line). The resonances at 66.2 ppm and 68.3 ppm were generated by the hyperpolarized 2-^{13}C nucleus of lactic acid.

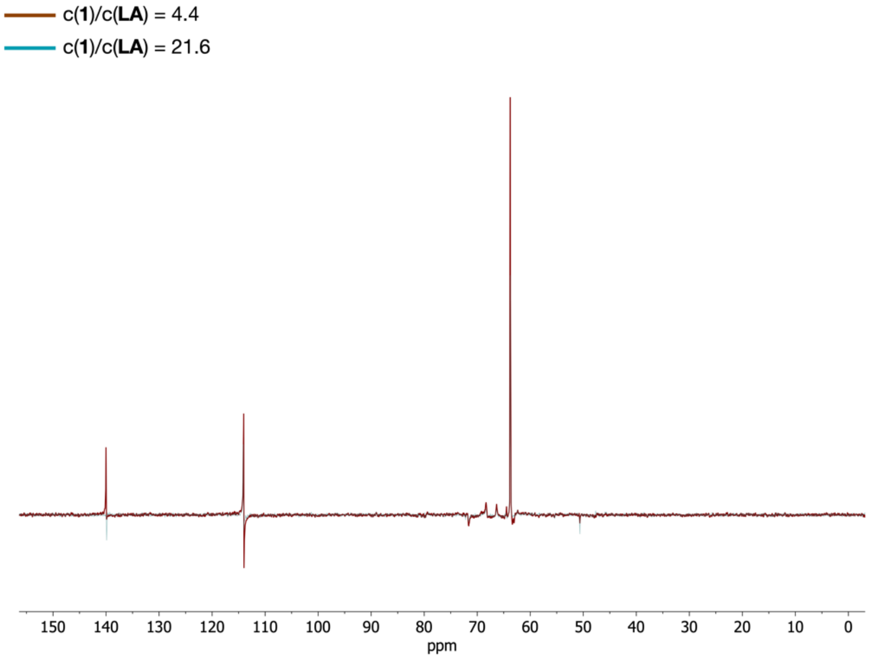

Figure S7: Superposition of ^{13}C-NMR spectra of $^{13}C_3$-lactic acid hyperpolarized using PHIP-X. The two corresponding PHIP-X experiments differ in c(1)/c(LA) =4.4 (brown line) and 21.6 (cyan line).

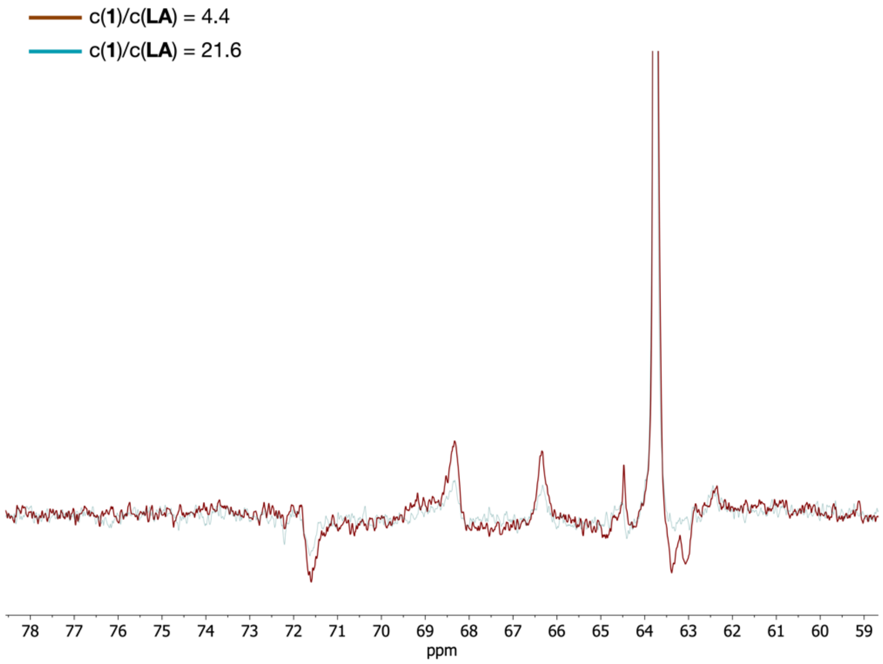

Figure S8: Superposition of ^{13}C-NMR spectra (zoom of figure S7) of $^{13}C_3$-lactic acid hyperpolarized using PHIP-X. The two corresponding PHIP-X experiments differ in c(1)/c(LA) =4.4 (brown line) and 21.6 (cyan line). The resonances at 66.2 ppm and 68.3 ppm were generated by the hyperpolarized 2-^{13}C nucleus of lactic acid.

^{13}C-NMR spectra of $^{13}C_3$-lactic acid hyperpolarized using PHIP-X (magnetic field dependence).

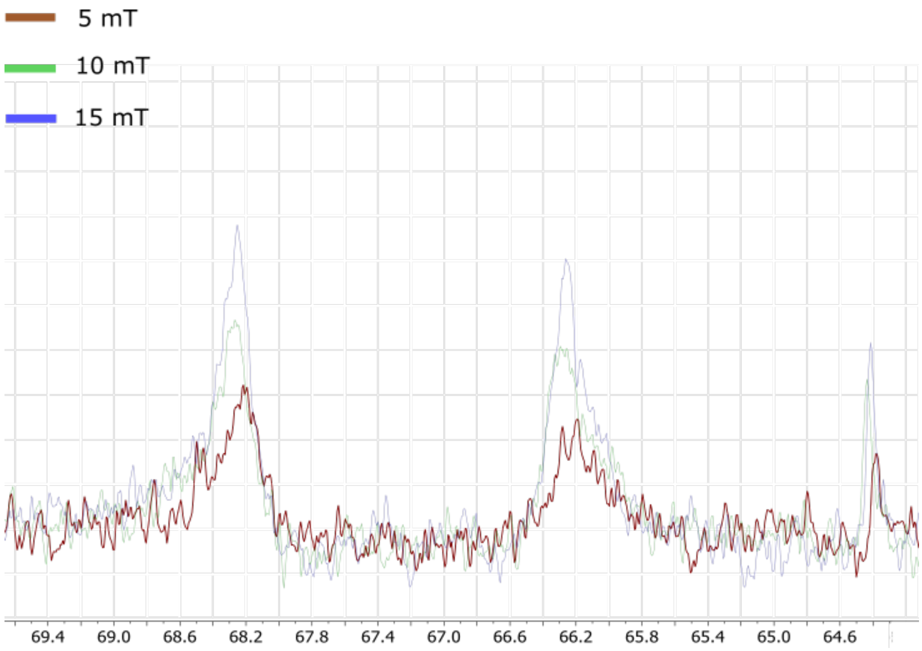

Figure S9: Superposition of ^{13}C-NMR spectra of $^{13}C_3$-lactic acid hyperpolarized using PHIP-X. The three corresponding PHIP-X experiments differ in B_{Pol0} = 5 (brown line), 10 (green line) and 15 (blue line) mT. The resonances at 66.2 ppm and 68.3 ppm were generated by the hyperpolarized 2-^{13}C nucleus of lactic acid.

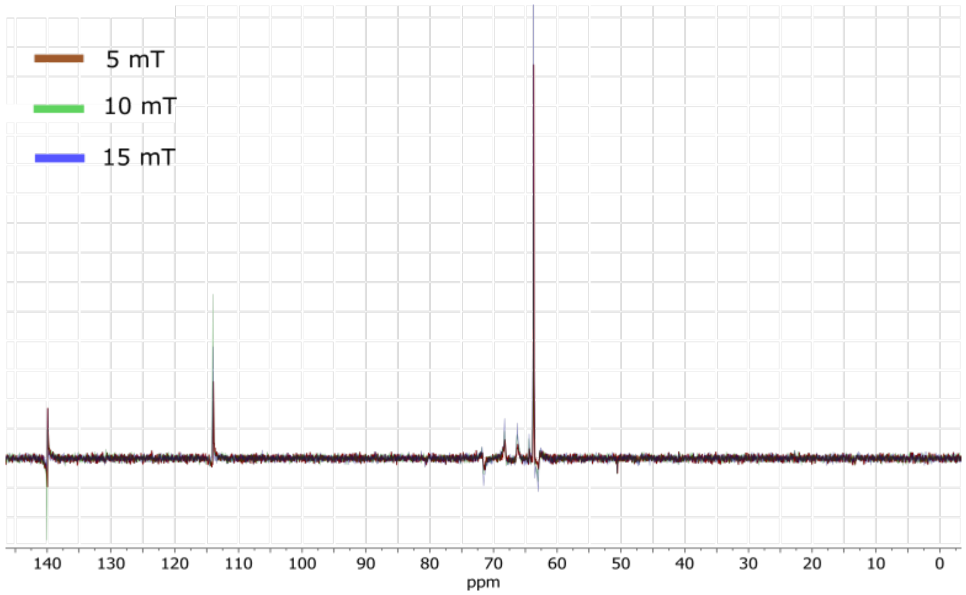

Figure S10: Superposition of ^{13}C-NMR spectra of $^{13}C_3$-lactic acid hyperpolarized using PHIP-X. The three corresponding PHIP-X experiments differ in B_{Pol0} = 5 (brown line), 10 (green line) and 15 (blue line) mT. The resonances at 66.2 ppm and 68.3 ppm were generated by the hyperpolarized 2-^{13}C nucleus of lactic acid. The three large resonances at 63.7, 114 and 140 ppm correspond to hyperpolarized allyl alcohol (the transfer agent).

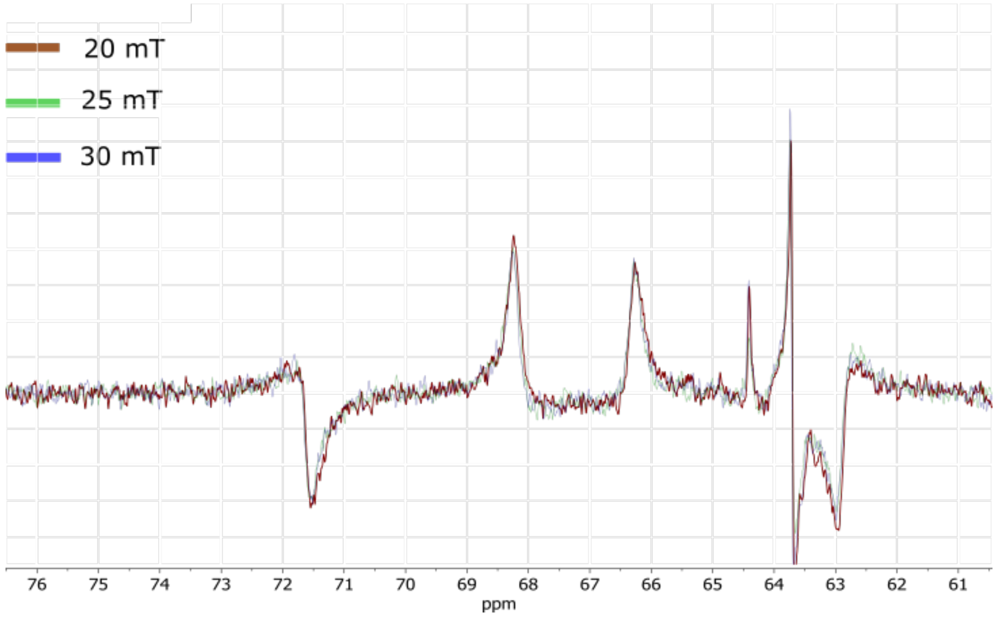

Figure S11: Superposition of ^{13}C-NMR spectra of $^{13}C_3$-lactic acid hyperpolarized using PHIP-X. The three corresponding PHIP-X experiments differ in B_{Pol0} = 20 (brown line), 25 (green line) and 30 (blue line) mT. The resonances at 63.0, 66.2, 68.3 and 71.5 ppm were generated by the hyperpolarized 2-^{13}C nucleus of lactic acid. The resonance at 63.7 ppm was generated by hyperpolarized allyl alcohol (the transfer agent).

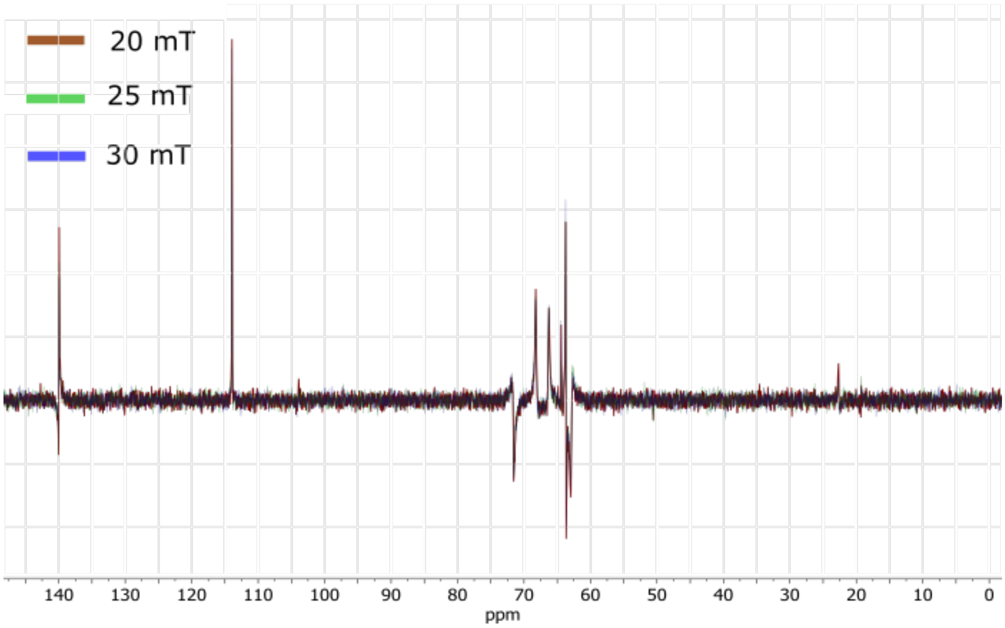

Figure S12: Superposition of ^{13}C-NMR spectra of $^{13}C_3$-lactic acid hyperpolarized using PHIP-X. The three corresponding PHIP-X experiments differ in B_{Pol0} = 20 (brown line), 25 (green line) and 30 (blue line) mT. The resonances at 63.0, 66.2, 68.3 and 71.5 ppm were generated by the hyperpolarized 2-^{13}C nucleus of lactic acid. The three strong resonances at 63.7, 114 and 140 ppm correspond to hyperpolarized allyl alcohol (the transfer agent).

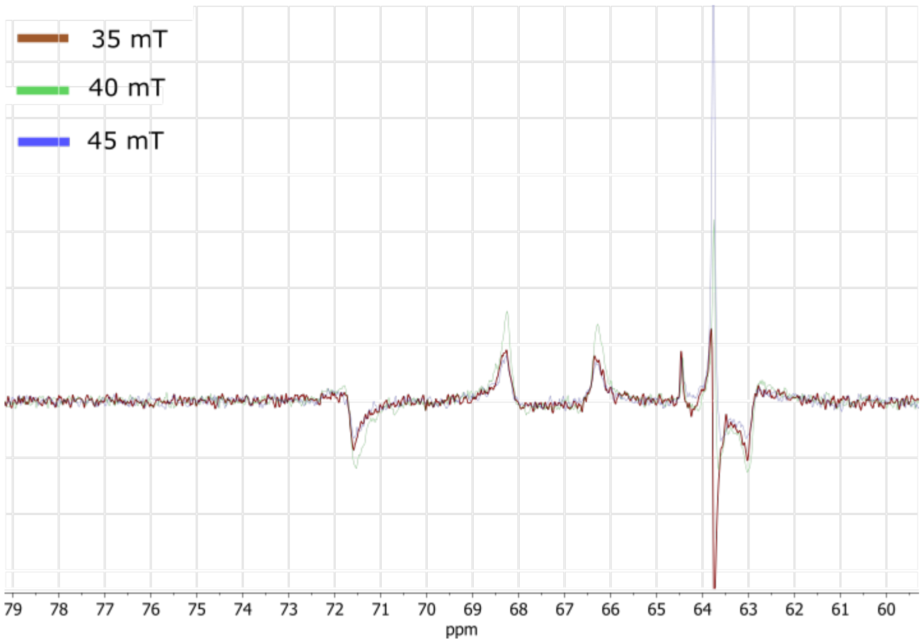

Figure S13: Superposition of ^{13}C-NMR spectra of $^{13}C_3$-lactic acid hyperpolarized using PHIP-X. The three corresponding PHIP-X experiments differ in B_{Pol0} = 35 (brown line), 40 (green line) and 45 (blue line) mT. The resonances at 63.0, 66.2, 68.3 and 71.5 ppm were generated by the hyperpolarized 2-^{13}C nucleus of lactic acid. The resonance at 63.7 was generated by hyperpolarized allyl alcohol (the transfer agent).

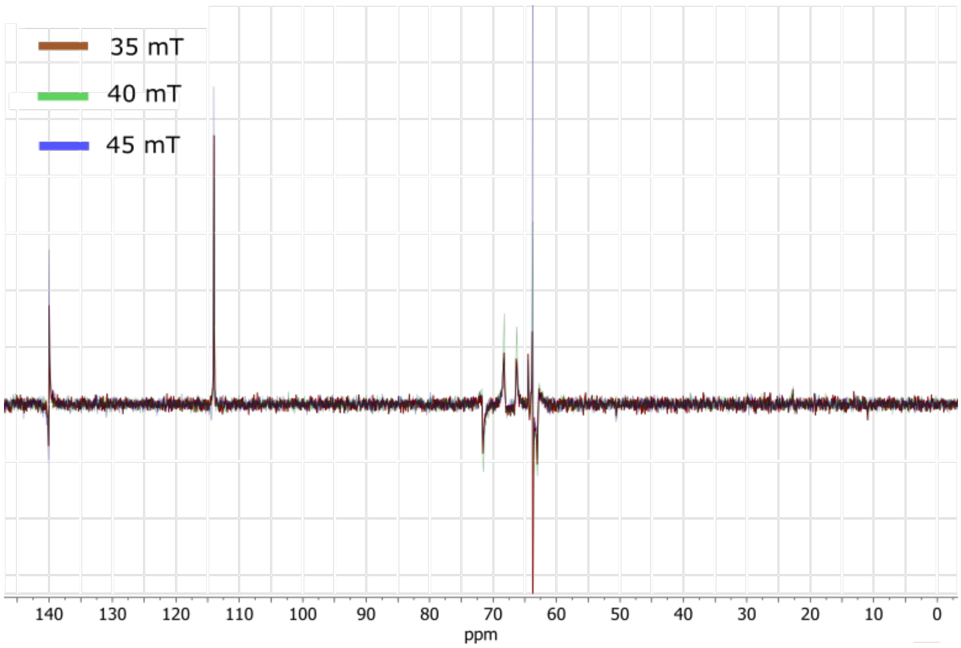

Figure S14: Superposition of ^{13}C-NMR spectra of $^{13}C_3$-lactic acid hyperpolarized using PHIP-X. The three corresponding PHIP-X experiments differ in B_{Pol0} = 35 (brown line), 40 (green line) and 45 (blue line) mT. The resonances at 63.0, 66.2, 68.3 and 71.5 ppm were generated by the hyperpolarized 2-^{13}C nucleus of lactic acid. The resonances at 63.7, 114 and 140 ppm were generated by hyperpolarized allyl alcohol (the transfer agent).

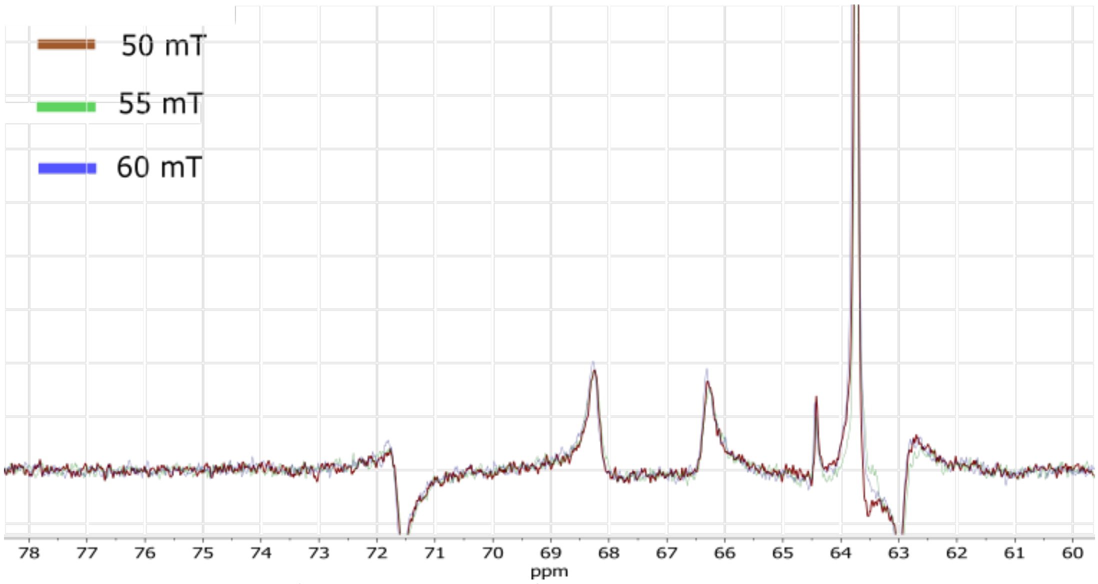

Figure S15: Superposition of ^{13}C-NMR spectra of $^{13}C_3$-lactic acid hyperpolarized using PHIP-X. The three corresponding PHIP-X experiments differ in B_{Pol0} = 50 (brown line), 55 (green line) and 60 (blue line) mT. The resonances at 63.0, 66.2, 68.3 and 71.5 ppm were generated by the hyperpolarized 2-^{13}C nucleus of lactic acid. The strong resonance at 63.7 was generated by hyperpolarized allyl alcohol (the transfer agent).

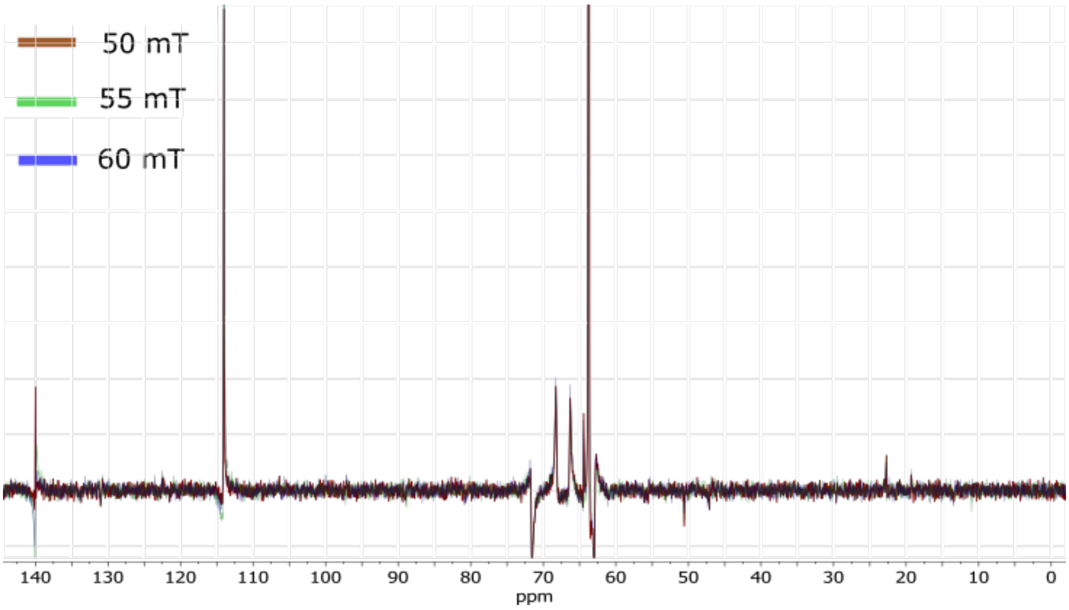

Figure S16: Superposition of ^{13}C-NMR spectra of $^{13}C_3$-lactic acid hyperpolarized using PHIP-X. The three corresponding PHIP-X experiments differ in B_{Pol0} = 50 (brown line), 55 (green line) and 60 (blue line) mT. The resonances at 63.0, 66.2, 68.3 and 71.5 ppm were generated by the hyperpolarized 2-^{13}C nucleus of lactic acid. The resonances at 63.7, 114 and 140 ppm were generated by hyperpolarized allyl alcohol (the transfer agent).

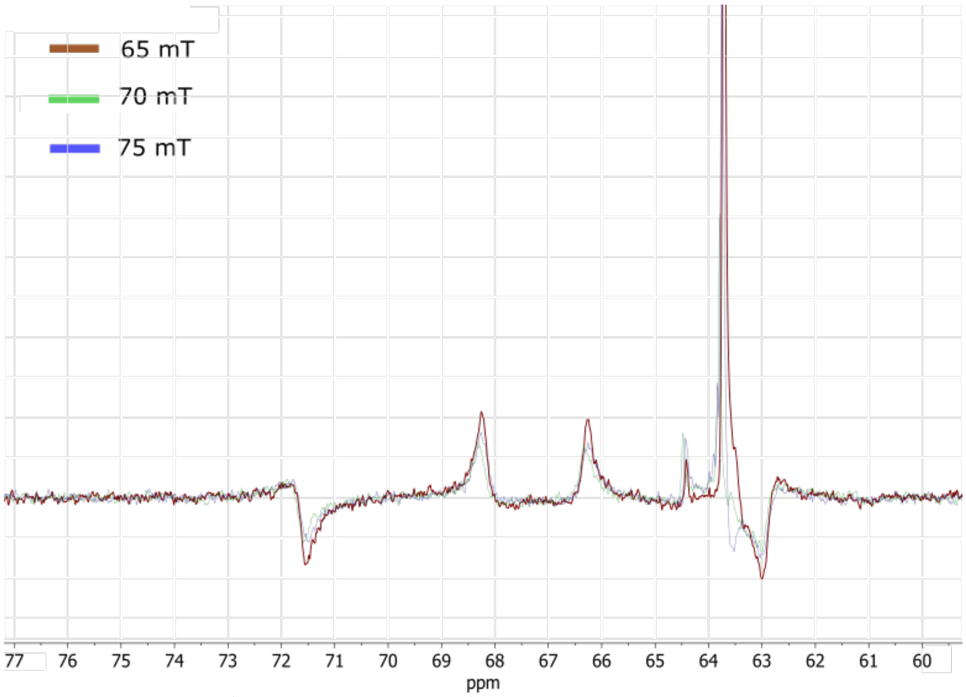

Figure S17: Superposition of ^{13}C-NMR spectra of $^{13}C_3$-lactic acid hyperpolarized using PHIP-X. The three corresponding PHIP-X experiments differ in B_{Pol0} = 65 (brown line), 70 (green line) and 75 (blue line) mT. The resonances at 63.0, 66.2, 68.3 and 71.5 ppm were generated by the hyperpolarized 2-^{13}C nucleus of lactic acid. The strong resonance at 63.7 was generated by hyperpolarized allyl alcohol (the transfer agent).

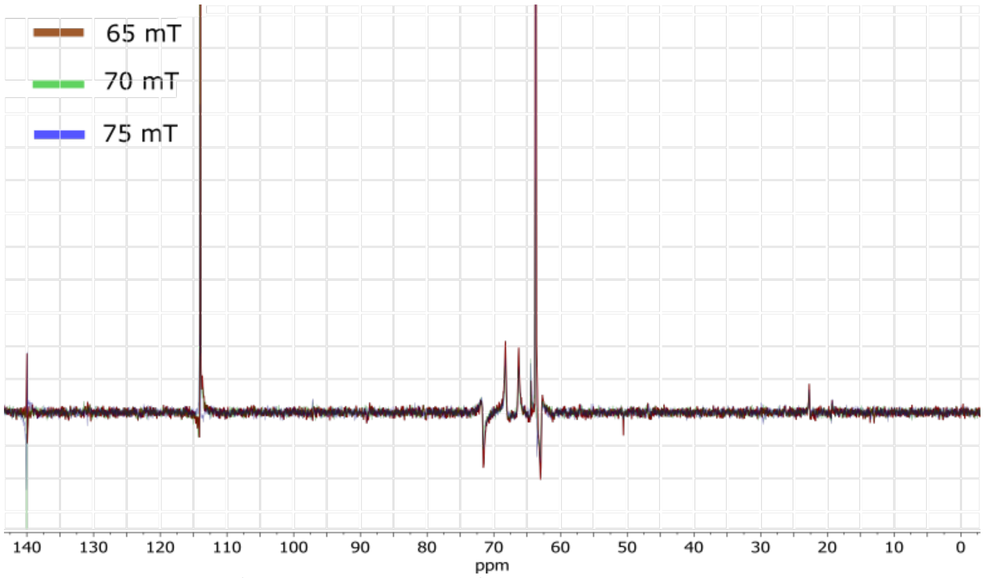

Figure S18: Superposition of ^{13}C-NMR spectra of $^{13}C_3$-lactic acid hyperpolarized using PHIP-X. The three corresponding PHIP-X experiments differ in B_{Pol0} = 65 (brown line), 70 (green line) and 75 (blue line) mT. The resonances at 63.0, 66.2, 68.3 and 71.5 ppm were generated by the hyperpolarized 2-^{13}C nucleus of lactic acid. The resonances at 63.7, 114 and 140 ppm were generated by hyperpolarized allyl alcohol (the transfer agent).

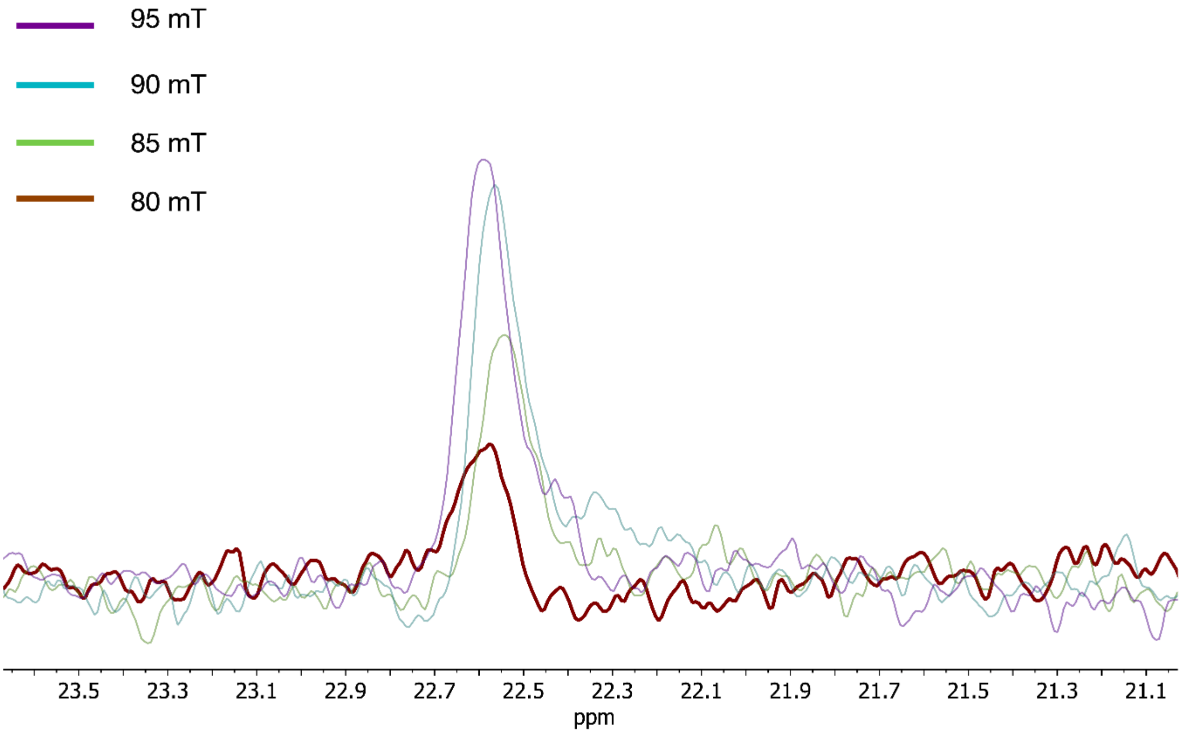

Figure S19: Superposition of ^{13}C-NMR spectra of $^{13}C_3$-lactic acid hyperpolarized using PHIP-X. The corresponding PHIP-X experiments differ in B_{Pol0} = 80 mT (brown line), 85 mT (green line), 90 mT (turquoise line) and 95 mT (violett line). The resonance at 22.6 ppm were generated by the hyperpolarized 3-^{13}C nucleus of lactic acid.

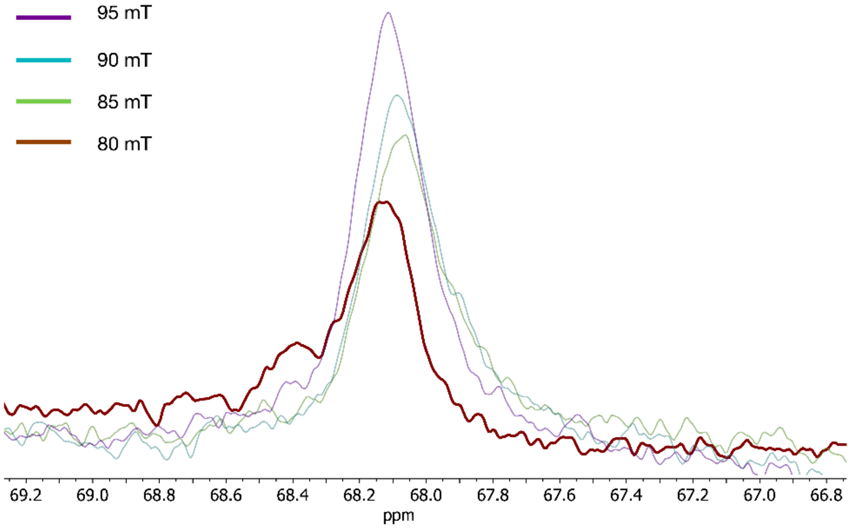

Figure S20: Figure S19: Superposition of ^{13}C-NMR spectra of $^{13}C_3$-lactic acid hyperpolarized using PHIP-X. The corresponding PHIP-X experiments differ in B_{Pol0} = 80 mT (brown line), 85 mT (green line), 90 mT (turquoise line) and 95 mT (violett line). The resonance at 68.1 ppm were generated by the hyperpolarized 2-^{13}C nucleus of lactic acid.

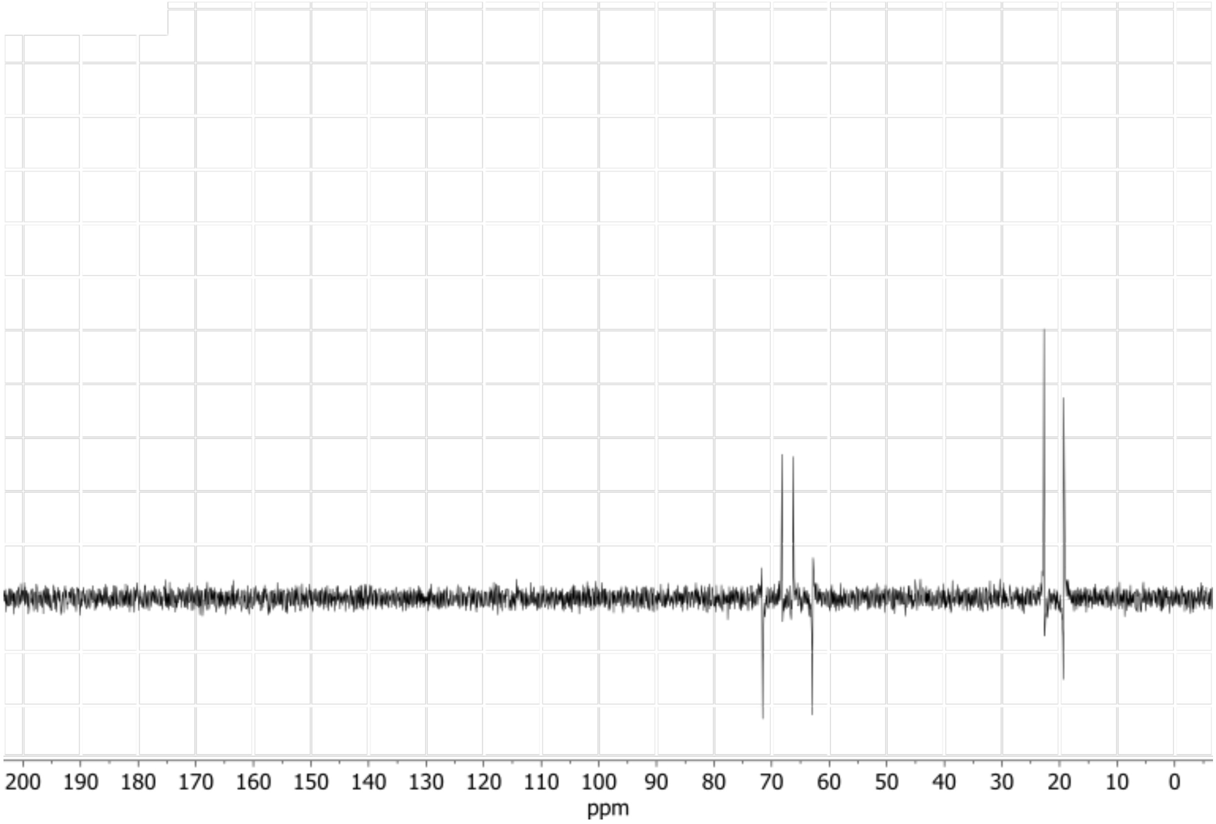

Figure S21: ^{13}C-NMR spectrum of thermal lactic acid. The spectrum was recorded using the same parameter as applied for the PHIP-X experiments (DEPT, 145 Hz), but with 1,000 scans instead of 1 scan. The resonances at 63.0, 66.2, 68.3 and 71.5 ppm were generated by the 2-^{13}C nucleus of thermal lactic acid. The two resonances around 20 ppm were generated by the 3-^{13}C nucleus of thermal lactic acid.

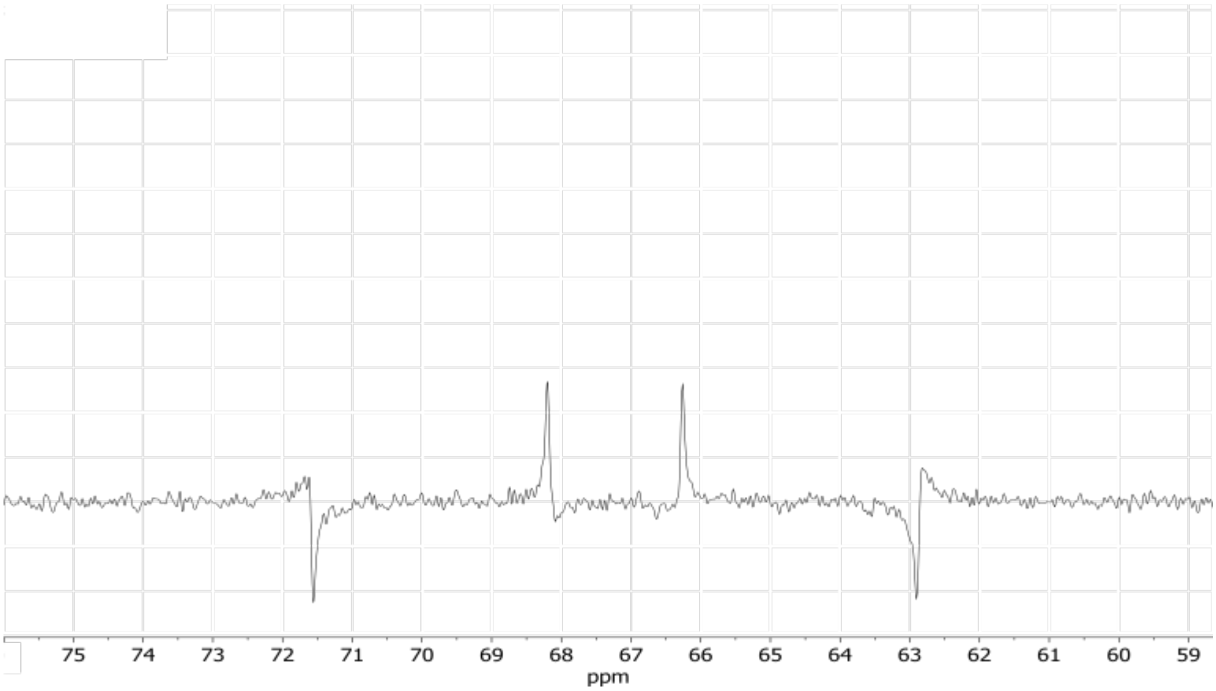

Figure S22: ^{13}C-NMR spectrum of thermal lactic acid. The spectrum was recorded using the same parameter as applied for the PHIP-X experiments (DEPT, 145 Hz), but with 1,000 scans instead of 1 scan. The resonances at 63.0, 66.2, 68.3 and 71.5 ppm were generated by the 2-^{13}C nucleus of thermal lactic acid.

^{13}C-NMR spectra of ^{13}C-Methanol hyperpolarized using PHIP-X.

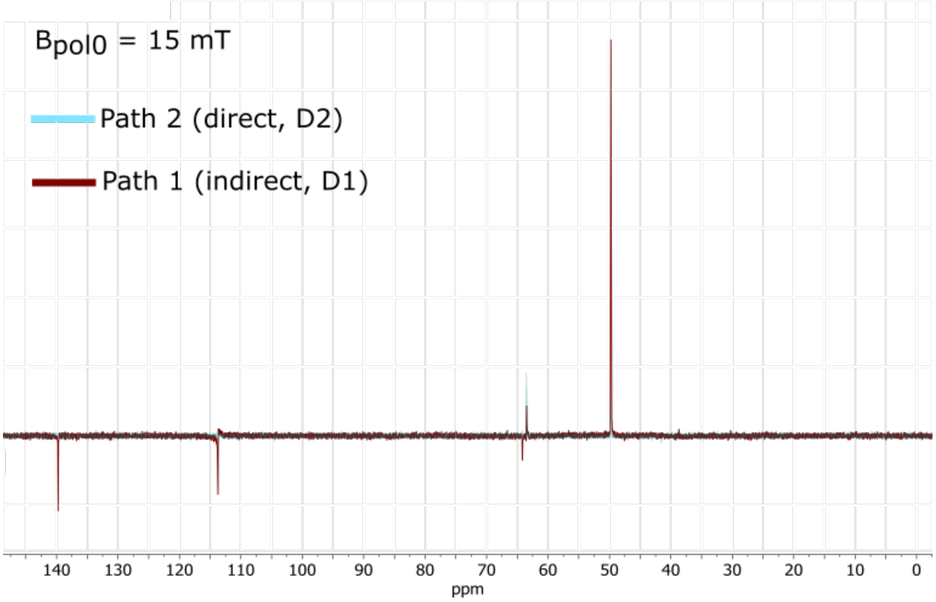

Figure S23: Superposition of two ^{13}C-NMR spectra of ^{13}C-methanol hyperpolarized using PHIP-X. The corresponding PHIP-X experiments differ in the pathways (D1 and D2 in fig. 1, main manuscript) how the polarization is transferred from the transfer agent (allyl alcohol) to the target ^{13}C-nucleus of methanol (at 49.77 ppm). Path 1 (brown line) provided stronger polarization yields and was selected by applying 139 Hz in the DEPT sequence. Path 2 (brown line) was selected by applying 3 Hz in the DEPT sequence. The resonances at 63.7, 114

and 140 ppm were generated by hyperpolarized allyl alcohol. The PHIP-X experiment was carried out at B_{Pol0} = 15 mT.

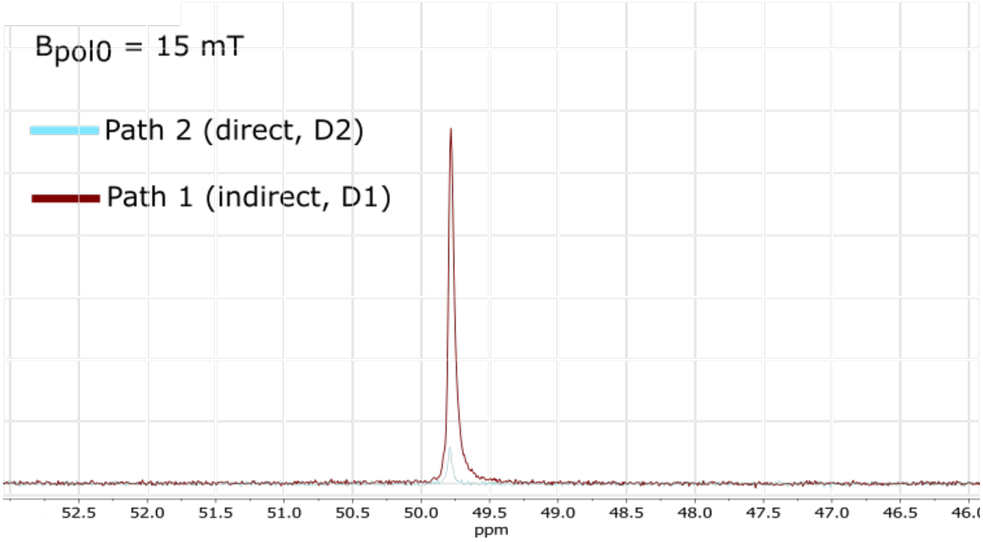

Figure S24: Superposition of two ^{13}C-NMR spectra of ^{13}C-methanol hyperpolarized using PHIP-X. The resonances at 49.77 Hz were generated by the hyperpolarized 13C-nucleus of methanol. The corresponding PHIP-X experiments differ in the pathways (D1 and D2 in fig. 1, main manuscript) how the polarization is transferred from the transfer agent (allyl alcohol) to the target ^{13}C-nucleus of methanol. Path 1 (brown line) provided stronger polarization yields and was selected by applying 139 Hz in the DEPT sequence. Path 2 (brown line) was selected by applying 3 Hz in the DEPT sequence. The PHIP-X experiment was carried out at B_{Pol0} = 15 mT.

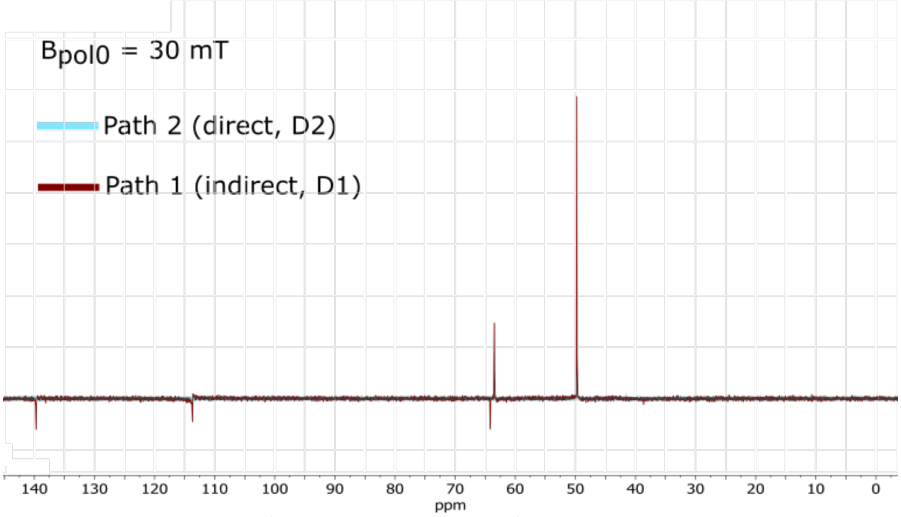

Figure S25: Superposition of two ^{13}C-NMR spectra of ^{13}C-methanol hyperpolarized using PHIP-X. The corresponding PHIP-X experiments differ in the pathways (D1 and D2 in fig. 1, main manuscript) how the polarization is transferred from the transfer agent (allyl alcohol) to the target ^{13}C-nucleus of methanol (at 49.77 ppm). Path 1 (brown line) provided stronger polarization yields and was selected by applying 139 Hz in the DEPT sequence. Path 2 (brown line) was selected by applying 3 Hz in the DEPT sequence. The resonances at 63.7, 114 and 140 ppm were generated by hyperpolarized allyl alcohol. The PHIP-X experiment was carried out at B_{Pol0} = 30 mT.

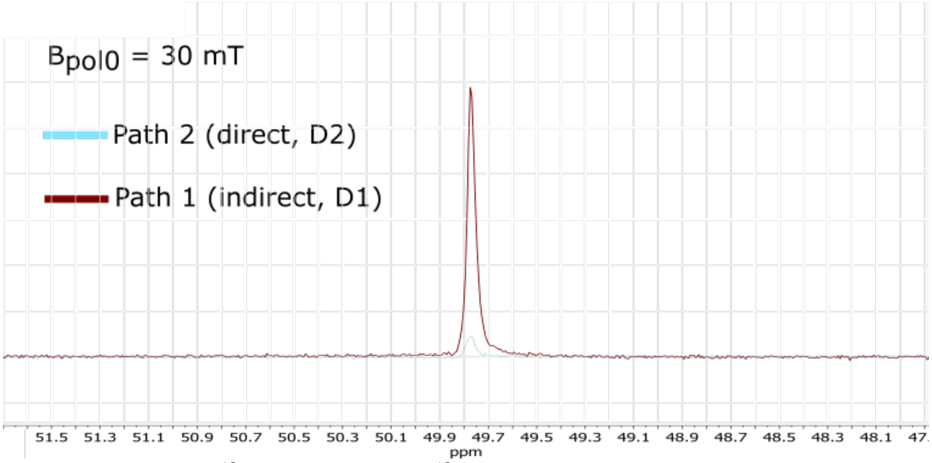

Figure S26: Superposition of two ^{13}C-NMR spectra of ^{13}C-methanol hyperpolarized using PHIP-X. The resonances at 49.77 Hz were generated by the hyperpolarized 13C-nucleus of methanol. The corresponding PHIP-X experiments differ in the pathways (D1 and D2 in fig. 1, main manuscript) how the polarization is transferred from the transfer agent (allyl alcohol) to the target ^{13}C-nucleus of methanol. Path 1 (brown line) provided stronger polarization yields and was selected by applying 139 Hz in the DEPT sequence. Path 2 (brown line) was selected by applying 3 Hz in the DEPT sequence. The PHIP-X experiment was carried out at B_{Pol0} = 30 mT.

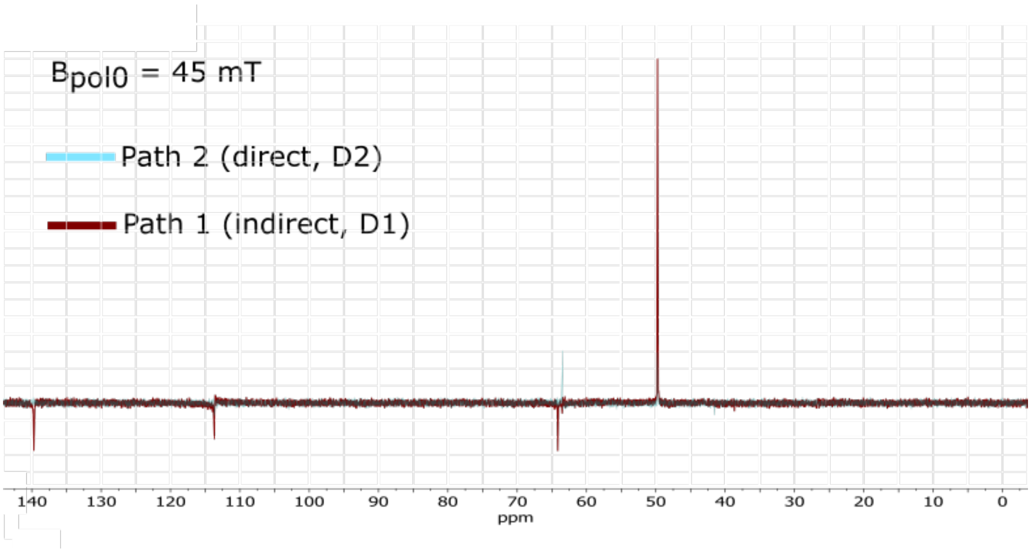

Figure S27: Superposition of two ^{13}C-NMR spectra of ^{13}C-methanol hyperpolarized using PHIP-X. The corresponding PHIP-X experiments differ in the pathways (D1 and D2 in fig. 1, main manuscript) how the polarization is transferred from the transfer agent (allyl alcohol) to the target ^{13}C-nucleus of methanol (at 49.77 ppm). Path 1 (brown line) provided stronger polarization yields and was selected by applying 139 Hz in the DEPT sequence. Path 2 (brown line) was selected by applying 3 Hz in the DEPT sequence. The resonances at 63.7, 114 and 140 ppm were generated by hyperpolarized allyl alcohol. The PHIP-X experiment was carried out at B_{Pol0} = 45 mT.

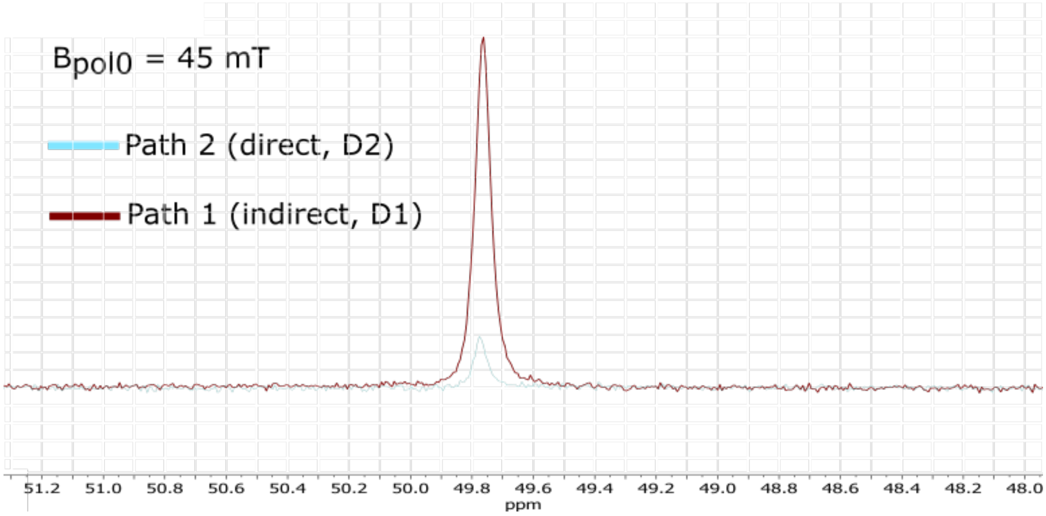

Figure S28: Superposition of two ^{13}C-NMR spectra of ^{13}C-methanol hyperpolarized using PHIP-X. The resonances at 49.77 Hz were generated by the hyperpolarized 13C-nucleus of methanol. The corresponding PHIP-X experiments differ in the pathways (D1 and D2 in fig. 1, main manuscript) how the polarization is transferred from the transfer agent (allyl alcohol) to the target ^{13}C-nucleus of methanol. Path 1 (brown line) provided stronger polarization yields and was selected by applying 139 Hz in the DEPT sequence. Path 2 (brown line) was selected by applying 3 Hz in the DEPT sequence. The PHIP-X experiment was carried out at B_{Pol0} = 45 mT.

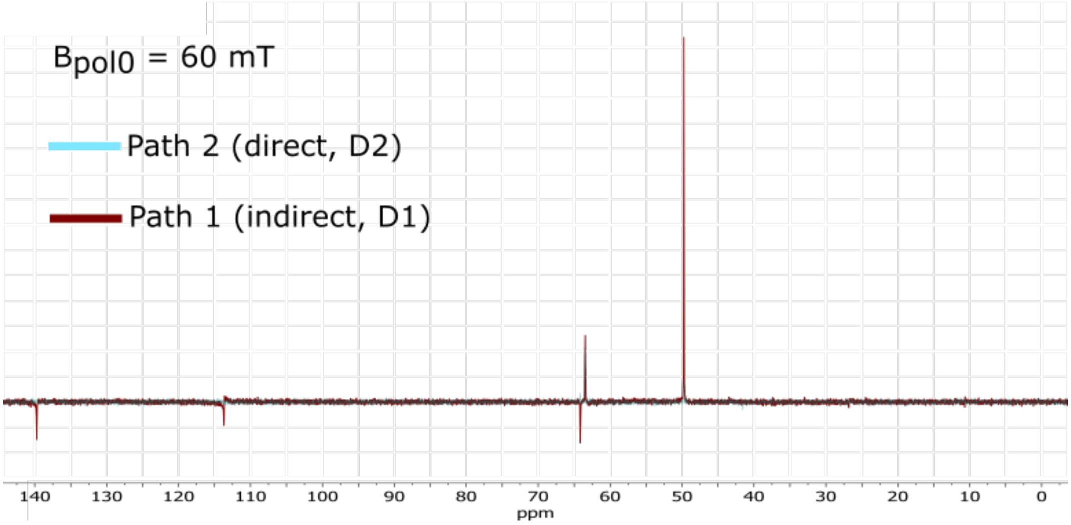

Figure S29: Superposition of two ^{13}C-NMR spectra of ^{13}C-methanol hyperpolarized using PHIP-X. The corresponding PHIP-X experiments differ in the pathways (D1 and D2 in fig. 1, main manuscript) how the polarization is transferred from the transfer agent (allyl alcohol) to the target ^{13}C-nucleus of methanol (at 49.77 ppm). Path 1 (brown line) provided stronger polarization yields and was selected by applying 139 Hz in the DEPT sequence. Path 2 (brown line) was selected by applying 3 Hz in the DEPT sequence. The resonances at 63.7, 114 and 140 ppm were generated by hyperpolarized allyl alcohol. The PHIP-X experiment was carried out at B_{Pol0} = 60 mT.

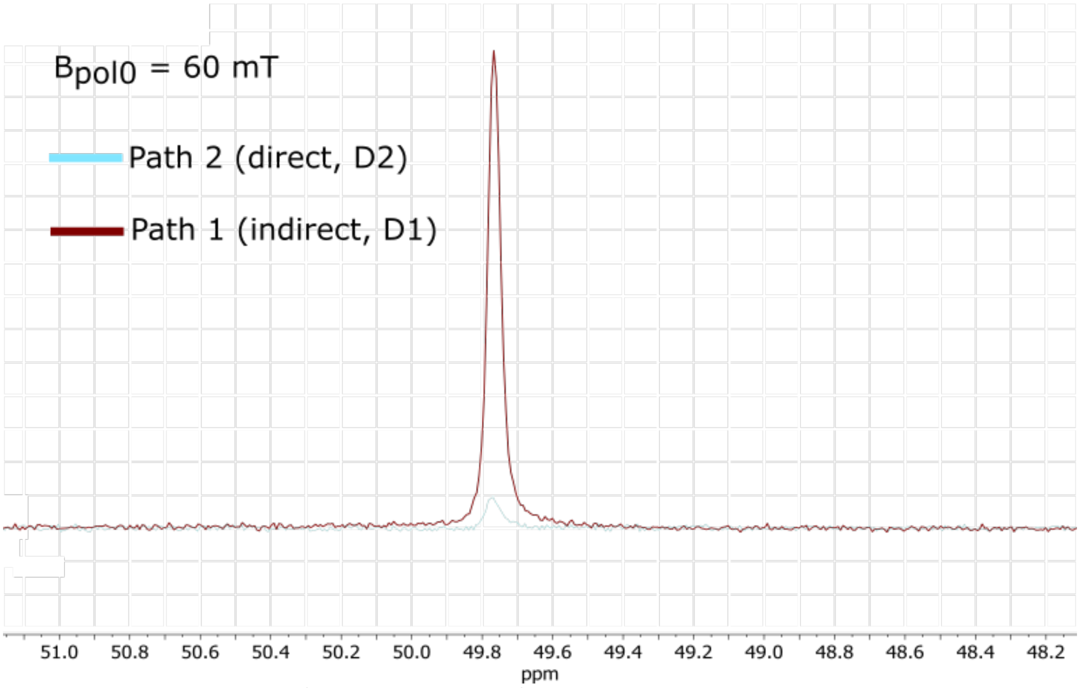

Figure S30: Superposition of two ^{13}C-NMR spectra of ^{13}C-methanol hyperpolarized using PHIP-X. The resonances at 49.77 Hz were generated by the hyperpolarized 13C-nucleus of methanol. The corresponding PHIP-X experiments differ in the pathways (D1 and D2 in fig. 1, main manuscript) how the polarization is transferred from the transfer agent (allyl alcohol) to the target ^{13}C-nucleus of methanol. Path 1 (brown line) provided stronger polarization yields and was selected by applying 139 Hz in the DEPT sequence. Path 2 (brown line) was selected by applying 3 Hz in the DEPT sequence. The PHIP-X experiment was carried out at B_{Pol0} = 60 mT.

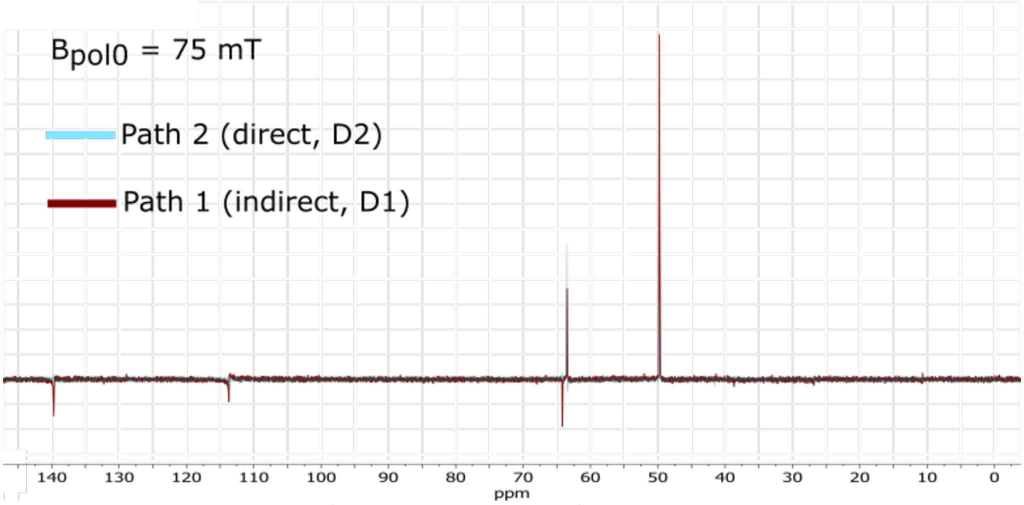

Figure S31: Superposition of two ^{13}C-NMR spectra of ^{13}C-methanol hyperpolarized using PHIP-X. The corresponding PHIP-X experiments differ in the pathways (D1 and D2 in fig. 1, main manuscript) how the polarization is transferred from the transfer agent (allyl alcohol) to the target ^{13}C-nucleus of methanol (at 49.77 ppm). Path 1 (brown line) provided stronger polarization yields and was selected by applying 139 Hz in the DEPT sequence. Path 2 (brown line) was selected by applying 3 Hz in the DEPT sequence. The resonances at 63.7, 114 and 140 ppm were generated by hyperpolarized allyl alcohol. The PHIP-X experiment was carried out at B_{Pol0} = 75 mT.

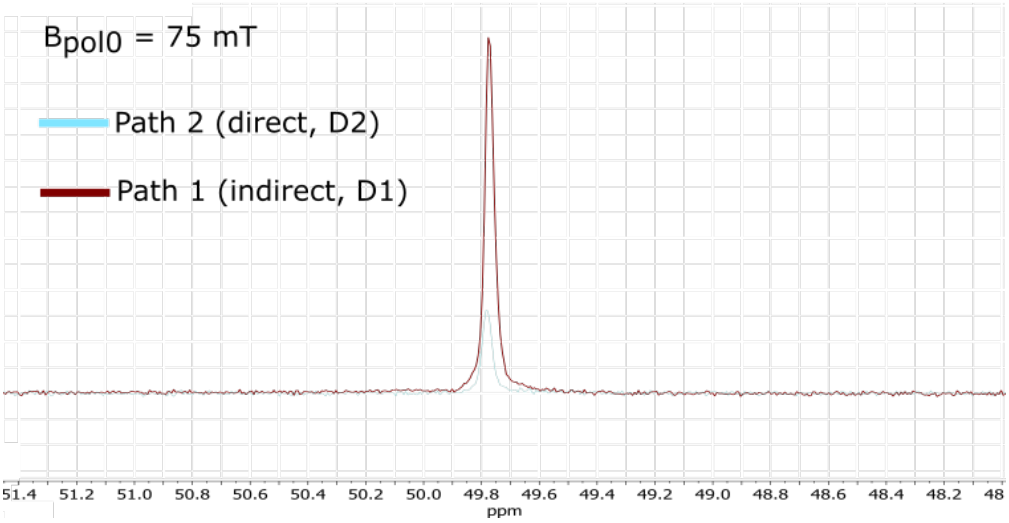

Figure S32: Superposition of two ^{13}C-NMR spectra of ^{13}C-methanol hyperpolarized using PHIP-X. The resonances at 49.77 Hz were generated by the hyperpolarized 13C-nucleus of methanol. The corresponding PHIP-X experiments differ in the pathways (D1 and D2 in fig. 1, main manuscript) how the polarization is transferred from the transfer agent (allyl alcohol) to the target ^{13}C-nucleus of methanol. Path 1 (brown line) provided stronger polarization yields and was selected by applying 139 Hz in the DEPT sequence. Path 2 (brown line) was selected by applying 3 Hz in the DEPT sequence. The PHIP-X experiment was carried out at B_{Pol0} = 75 mT.

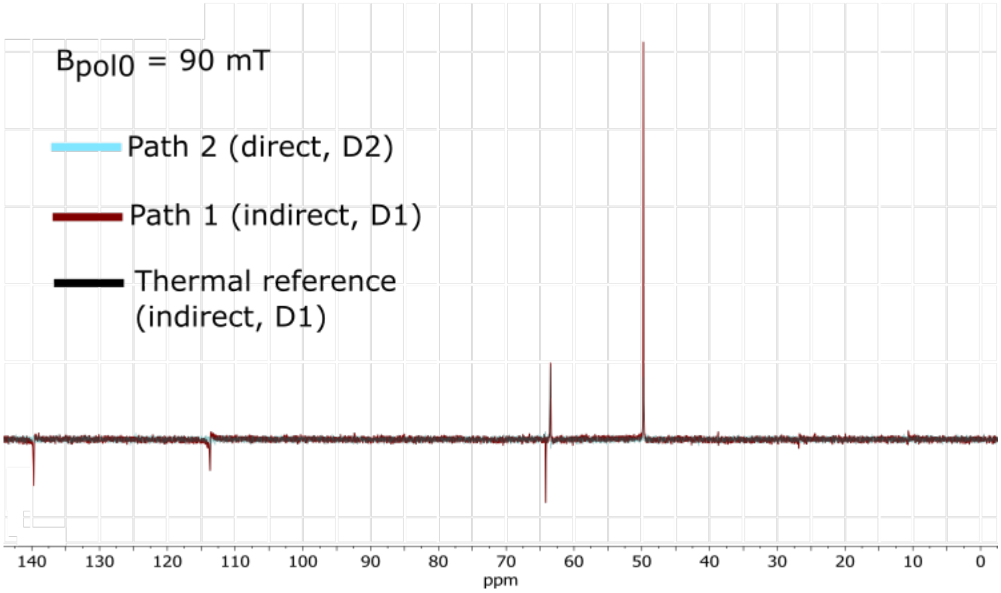

Figure S33: Superposition of two ^{13}C-NMR spectra of ^{13}C-methanol hyperpolarized using PHIP-X. The corresponding PHIP-X experiments differ in the pathways (D1 and D2 in fig. 1, main manuscript) how the polarization is transferred from the transfer agent (allyl alcohol) to the target ^{13}C-nucleus of methanol (at 49.77 ppm). Path 1 (brown line) provided stronger polarization yields and was selected by applying 139 Hz in the DEPT sequence. Path 2 (brown line) was selected by applying 3 Hz in the DEPT sequence. The resonances at 63.7, 114 and 140 ppm were generated by hyperpolarized allyl alcohol. The PHIP-X experiment was carried out at B_{Pol0} = 90 mT.

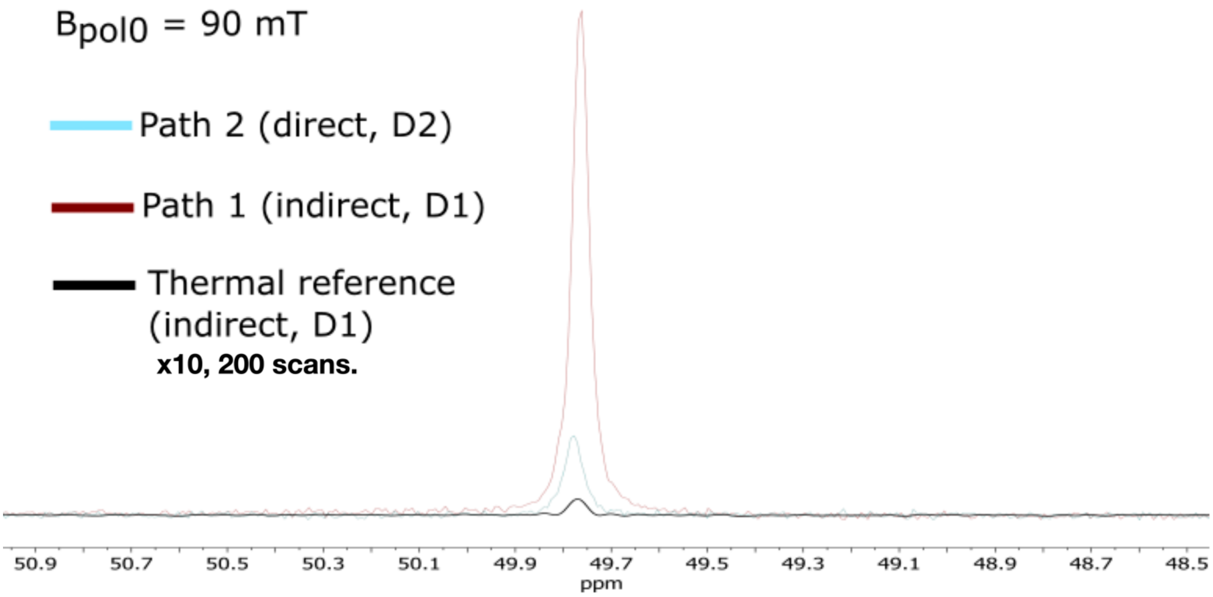

Figure S34: Superposition of two ^{13}C-NMR spectra of ^{13}C-methanol hyperpolarized using PHIP-X. The resonances at 49.77 Hz were generated by the hyperpolarized 13C-nucleus of methanol. The thermal reference was recorded 30 minutes after the PHIP-X experiment using the same sample. The spectrum of the thermal reference is enlarged by a factor of 10 and recorded using 200 scans. The corresponding PHIP-X experiments differ in the pathways (D1 and D2 in fig. 1, main manuscript) how the polarization is transferred from the transfer agent (allyl alcohol) to the target ^{13}C-nucleus of methanol. Path 1 (brown line) provided stronger polarization yields and was selected by applying 139 Hz in the DEPT sequence. Path 2 (brown line) was selected by applying 3 Hz in the DEPT sequence. The PHIP-X experiment was carried out at B_{Pol0} = 90 mT.

^{1}H-NMR spectra of ^{13}C-Methanol hyperpolarized using PHIP-X.

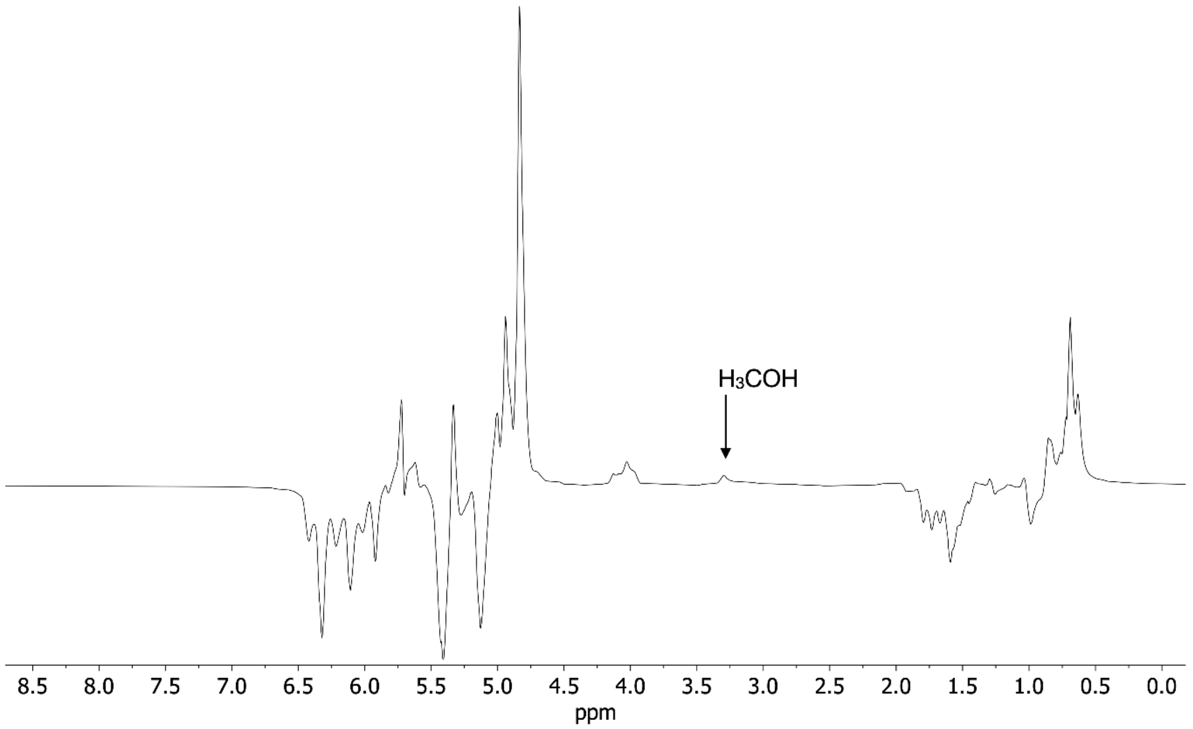

Figure S35: ^{1}H NMR spectrum of methanol hyperpolarized using PHIP-X. The signal of the hyperpolarized methyl group of methanol is located at 2.29 ppm and marked by an arrow. The corresponding signal gain is about 300-fold (P=0.1%) compared to the thermal spectrum (figure S36).

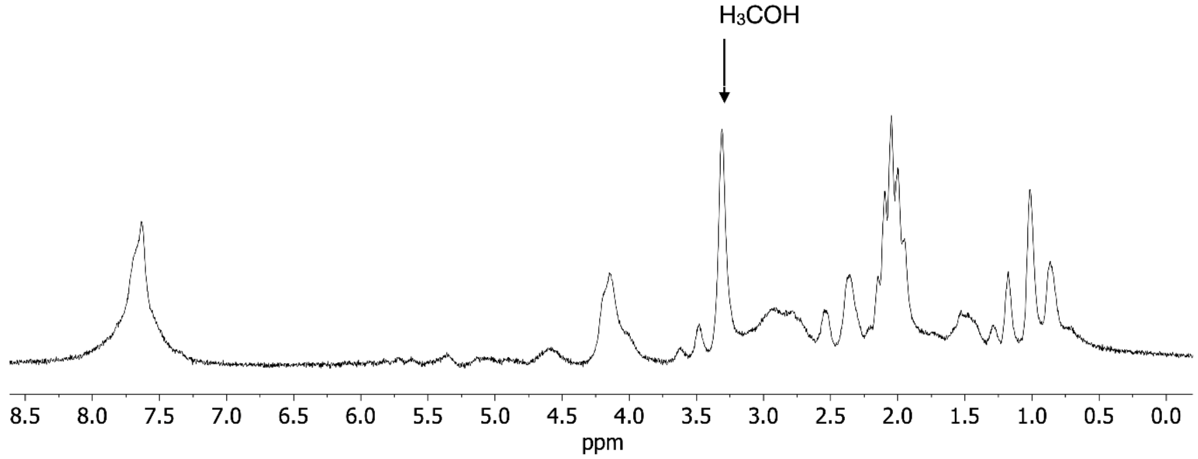

Figure S35: ^{1}H NMR spectrum recorded after thermalization of the solution which was used to hyperpolarize methanol using PHIP-X. The thermal signal of the methyl group of methanol is located at 2.29 ppm and marked by an arrow.

^{13}C-NMR spectra of hyperpolarized styrene in the presence of methanol.

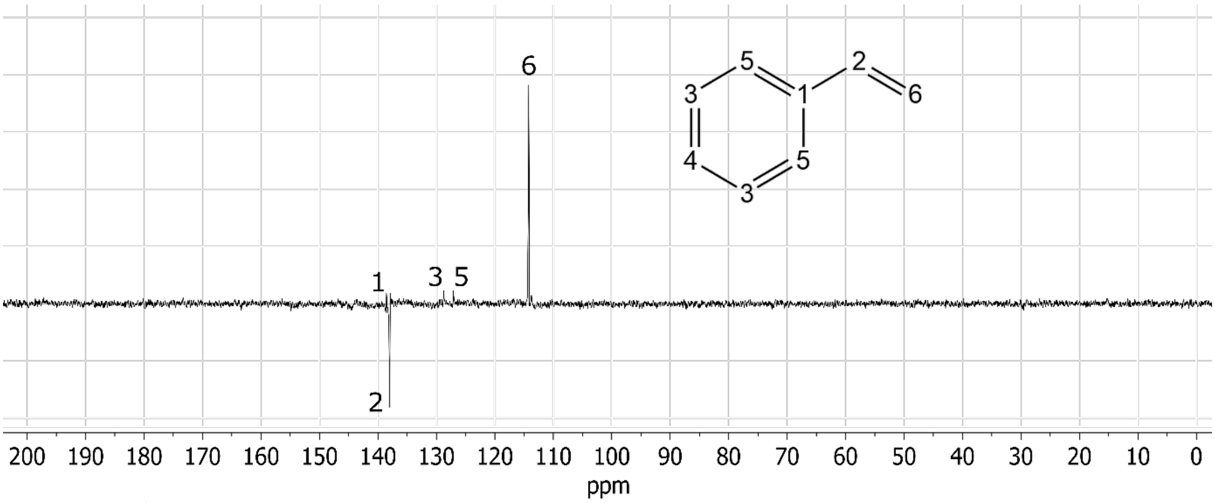

Figure S36: ^{13}C NMR spectrum of hyperpolarized styrene in acetone-d6 after a PHIP-X. The solution contained 13C-methanol to check if there is a polarization transfer from styrene to methanol. All other parameters like pH_2-pressure were the same as in the PHIP-X experiments containing propargyl alcohol. B_{Pol0} was set to 90 mT. However, no polarization transfer was detected when using phenylacetylene. This is in contrast to the experiments containing propargyl alcohol, where strong 13C polarization of methanol was observed. One may interpret this result as a hint that labile protons mediate the polarization transfer in PHIP-X.

Spin dynamics simulations.

We analyzed the dependence of the target polarizations (^{1}H in Fig. S37b and ^{13}C in c) for different polarization levels of the labile protons (Fig. S37a) and found a linear dependence (Fig. S37).

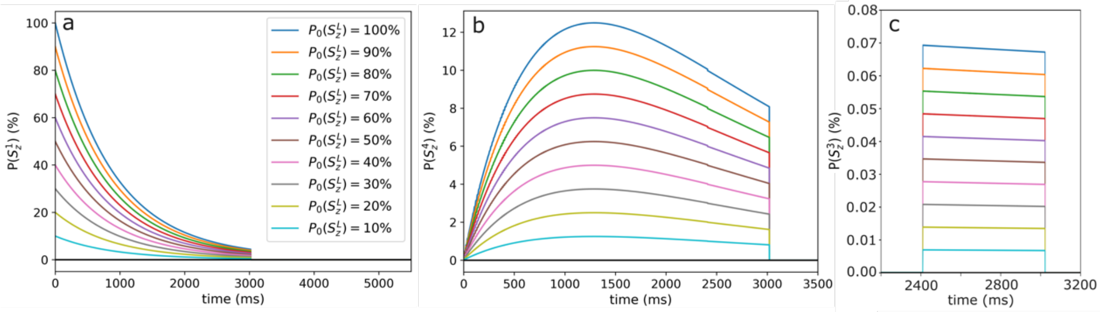

Figure S37: The target polarizations (^{1}H in b and ^{13}C in c) depend linear on the polarization level of the labile protons (a). MFC is applied at 2,400 ms and a simple 90° pulse is applied at 3,030 ms. The simulation parameters are set to B_{Pol0} = 90 mT, K_1 = K_2 = 200 1/s, T_1 = 1 s for the labile protons (No. 1 and 2), T_1 = 20 s for the ^{13}C nucleus (spin No. 3) and T_1 = 4 s for the fixed target proton (spin No. 4). We have $P(S_Z^1)$(t) = $P(S_Z^2)$(t) for all times t.
